# Organization of Excitable Dynamics in Hierarchical Biological Networks

**DOI:** 10.1371/journal.pcbi.1000190

**Published:** 2008-09-26

**Authors:** Mark Müller-Linow, Claus C. Hilgetag, Marc-Thorsten Hütt

**Affiliations:** 1 Bioinformatics Group, Department of Biology, Darmstadt University of Technology, Darmstadt, Germany; 2 School of Engineering and Science, Jacobs University Bremen, Bremen, Germany; Indiana University, United States of America

## Abstract

This study investigates the contributions of network topology features to the dynamic behavior of hierarchically organized excitable networks. Representatives of different types of hierarchical networks as well as two biological neural networks are explored with a three-state model of node activation for systematically varying levels of random background network stimulation. The results demonstrate that two principal topological aspects of hierarchical networks, node centrality and network modularity, correlate with the network activity patterns at different levels of spontaneous network activation. The approach also shows that the dynamic behavior of the cerebral cortical systems network in the cat is dominated by the network's modular organization, while the activation behavior of the cellular neuronal network of *Caenorhabditis elegans* is strongly influenced by hub nodes. These findings indicate the interaction of multiple topological features and dynamic states in the function of complex biological networks.

## Author Summary

Many complex biological networks are characterized by the coexistence of topological features such as modules and central hub nodes. What are the relative contributions of these structural features to the networks' dynamic behavior? We used a computational model to simulate the general activation and inactivation behavior of excitable nodes in neural networks and studied the spread of activity in hierarchically organized networks as well as specific biological neural networks. We then evaluated the impact of modules and hub nodes on the network dynamics by correlating the patterns of node activity with the network architecture at difference levels of spontaneous network activation. Two dynamic regimes were observed: waves propagating from central nodes and module-based synchronization. Remarkably, the dynamic behavior of hierarchical modular networks switched between these modes as the level of spontaneous activation changed. We also found that the two dynamic regimes have different significance in the neuronal network of *C. elegans*, where activity is mainly organized by hub nodes, and the systems network of the cat cerebral cortex, which is dominated by the network's modular organization. Our approach can be used to dynamically explore the organization of complex neural networks, beyond the structural characterizations that were available previously.

## Introduction

### Hierarchical Biological Networks

The analysis of biological networks presents an intriguing challenge, due to the complex, non-random organization of these systems and the diverse dynamic behaviors that they express. The topology of several biological networks has been shown to be based on a scale-free degree distribution, which implies the existence of highly connected network hubs [1],[2]. Biological systems were also found to be organized in network modules [3],[4], or to contain characteristic circuits (motifs) that do not occur as frequently in other types of networks [5]. Hub nodes, which have been identified in several biological networks, such as protein-protein interaction networks or metabolic networks, may serve as central distributing elements or linkage point for many regions of a network [2],[6],[7]. Such hubs might also be present in neural systems networks [8]. A hub, for our purposes, can either be a node with a high degree or with a high centrality (i.e. with many shortest paths between nodes passing through). For our purposes, the latter definition is dynamically more relevant. Modules or network clusters, which are characterized by a higher frequency or density of connections within than between node clusters [9] have been identified in biological metabolic networks [10],[11], as well as neural networks at the cellular level [12] or the systems level [13]. These modules often represent a specific function, e.g. a specific synthesis pathway in a metabolic reaction network [14], and may shape the functional interactions within the networks at different scales [15]–[17]. It has also been argued that motifs may represent specific functional circuits [18]–[20].

In addition to the mentioned features, the organization of biological systems is often described as hierarchical. However, no formal definition of hierarchical topology appears to exist. Typical descriptions of hierarchical organization use a modules-within-modules view [10],[21], others focus on the coexistence of modules and central (hub) nodes [11],[22] or relate the concept of hierarchy to fractality [23]. The distinction between hubs which organize modules around them and hubs which connect modules on a higher topological level has been productive for understanding the functional roles of these hub categories in various empirical networks [11],[22],[24]. Note: (1) In [10] the algorithm for generating modules within modules, leading to a hierarchical network, also produces a hierarchy of hubs in the network; (2) it is not immediately clear, whether the fractal graphs discussed in [23] are also “fractal” from the perspective of the box-counting formalism developed in [25],[26]. Particularly the latter concept of fractality has interesting implications for the organization of dynamic processes on the graph [27].

In the present paper, we attempt to summarize current topological concepts, condense the spectrum of different network arrangements into a few salient topological features and, using a simple three-state model of excitable dynamics on graphs, study how these topological features organize dynamic behavior. While this approach and our findings are valid for a wide range of networks, we investigate the question and the implications of our findings particularly in the context of neural networks, which most clearly express diverse patterns of excitable dynamics.

### Models of Network Topology

**Figure 1:**
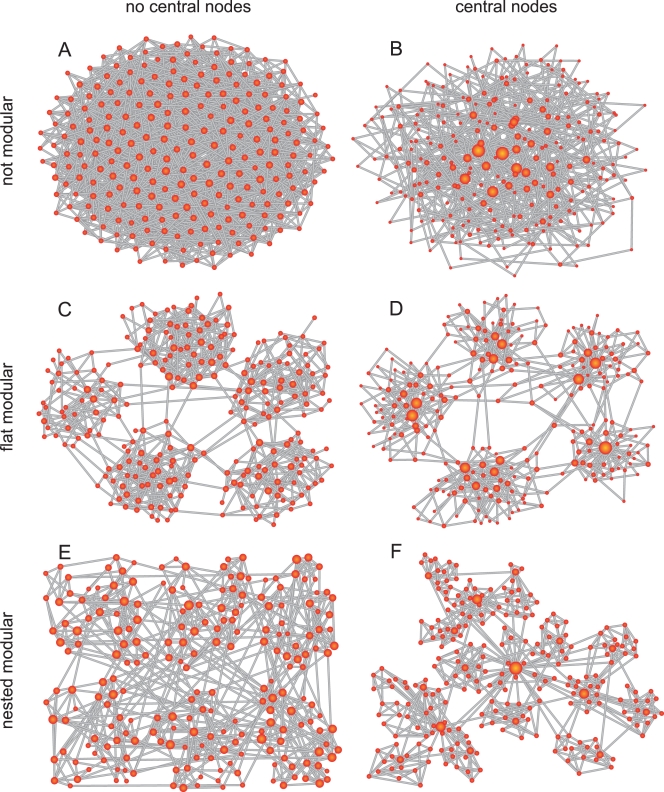
Basic graph models representing different combinations of both modular and hub characteristics. The degree of a node (as an example of a hub characteristic) is indicated by its size, while the grouping of the nodes reveals the modular structure. (A) The Erdös-Rényi (ER) random graph lacks both hubs and modules; (B) the scale-free Barabási-Albert (BA) graph displays a center of interlinked hubs only; the (C) random modular graph and the (D) scale-free modular graph consist of planarly linked modules, which are composed of smaller ER graph and BA graphs, respectively. The hubs in the BA graph version are distributed among the modules. The hierarchical graphs in (E) and (F) are featured by modules consisting of modules. In contrast to the hierarchical cluster graph in (E), the hierarchical scale-free graph (F) is additionally characterized by a hierarchical structure of hubs with one hub dominating the center.

From a combination of modular and hub features, various types of network topologies can arise. Classical Erdös-Rényi (ER) random graphs do not contain hubs or modules and may thus serve as a general null model. Scale-free Barabási-Albert (BA) graphs, on the other hand, contain only hubs and no modules. Within such graphs, projections from the hubs can reach many network regions, and the hub nodes thus have a more privileged role than nodes with fewer connections and a more restricted reach. On the other hand, networks that do not contain hubs, but are modular, may arise from linking many distributed, dense clusters with a small number of inter-cluster connections. Such clusters could exist at different levels (representing clusters of sub-clusters of sub-sub-clusters [21]), resulting in a hierarchical network organization, which has recently been termed “fractal” [23]. Finally, networks may be modular and also contain hubs, which are either contained within the modules serving as local hubs, or may form global hubs that integrated network modules at different scales of organization [10],[14],[24],[28]. The two latter networks combine features of scale-free and modular networks. Figure 1 summarizes the topology of modular and hub features and their combination in complex networks. While all feature combinations provide networks of complex organization, we are particular interested in the hierarchical networks shown in the last row of Figure 1, which form modular arrangements, with or without hubs, at different network scales.

### Models of Network Dynamics

For discussing the link between network topology and dynamics we use a simple three-state model of an excitable medium. The model consists of three discrete states for each node (susceptible *S*, excited *E*, refractory *R*), which are updated synchronously in discrete time steps according to the following rules: (1) A susceptible node becomes an excited node, if there is at least one excitation in its direct neighborhood. If not, spontaneous firing occurs with the probability *f*, which is the rate of spontaneous excitation; (2) an excited node enters the refractory state; (3) a node regenerates (*R*→*S*) with the recovery probability *p* (the inverse of which is the average refractory time of a node). This minimal model of an excitable system has a rich history in biological modeling. It has been first introduced in a simpler variant under the name “forest fire model” [29] and subsequently expanded by Drossel and Schwabl [30] who also introduced the rate of spontaneous excitations (the “lightning probability” in their terminology). In this form it was originally applied on regular architectures in studies of self-organized criticality. Other variants of three-state excitable dynamics have been used to describe epidemic spreading [31]–[34]. As discussed previously [35],[36], this general model can readily be implemented on arbitrary network architectures. It has been shown that short-cuts inserted into a regular (e.g., ring-like) architecture can mimic the dynamic effect of spontaneous excitations [35]. Using a similar model setup we have recently shown [36] that the distribution pattern of excitations is regulated by the connectivity as well as by the rate of spontaneous excitations. An increase in each of these two quantities leads to a sudden increase in the excitation density accompanied by a drastic change in the distribution pattern from a collective, synchronous firing of a large number of nodes in the graph (spikes) to more local, long-lasting and propagating excitation patterns (bursts). Further studies on the activity of integrate-and-fire neurons in the classical small-world model from [37] also revealed a distinct dependency of the dynamic behavior on the connectivity of the system [38].

Here, we take this investigation one step further by analyzing which topological properties determine the distribution patterns of excitations. In order to study these patterns, we consider the individual time series of all nodes and for each pair of nodes (*s*,*t*) compute the number *C* = *C_st_* of simultaneous firing events. When applied to the whole network the resulting matrix *C* essentially represents the distribution pattern of excitations which we now can compare with a corresponding distribution pattern of some topological property.

Examining hub and modular aspects of topology separately we first investigate which of them explains best the observed pattern of simultaneous firing events. In particular, we show that in different parameter regimes (characterized by the rate of spontaneous excitations) different topological properties determine the observed synchronization patterns. Moreover, we show that small systematic changes in the graph architecture, designed to enhance or decrease the selected topological property, are reflected in the dynamics. In a second step, we extend our study to hierarchically structured artificial graphs and then to biological networks, in order to demonstrate that the distribution patterns of excitations change dramatically when both properties are represented to different degrees in the respective graphs. Finally, we summarize our results and discuss limitations of the present approach, and extend our observations to describe general principles of pattern formation on graphs.

## Results

### Overview

**Figure 2:**
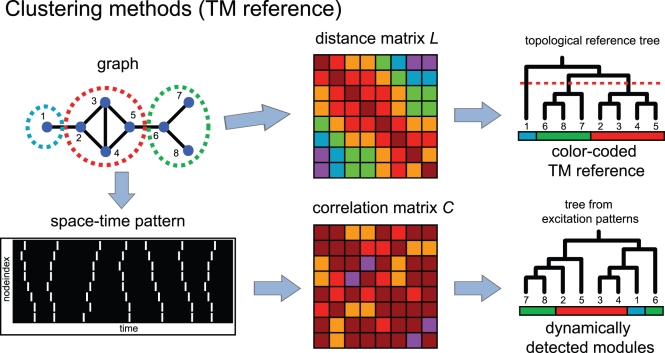
Construction of a color-coded topological reference based on the TM of a network (Top), and formation of a dynamic clustering tree on the basis of the dynamic model simulation (Bottom). (Top) The distance relations between all nodes are converted into a distance matrix *L*. (The color label encodes the distances between pairs of nodes.) The matrix *L* is then translated into a topological reference tree via UPGMA clustering (see Methods). The node indices in the graph correspond to the ones in the tree, the circles in the graph representation denote the modules found in the cluster tree after assigning a threshold (dotted line) which separates the downstream branches. Next, color labels are assigned (TM reference). (Bottom) The model produces a space-time pattern of excitations (white lines) which is then converted into a correlation matrix *C*. (The color labels encode the number of simultaneous excitations.) The matrix *C* is translated into a clustering tree (from the excitation patterns). The color labels of the leaves are copied from the TM reference.

**Figure 3:**
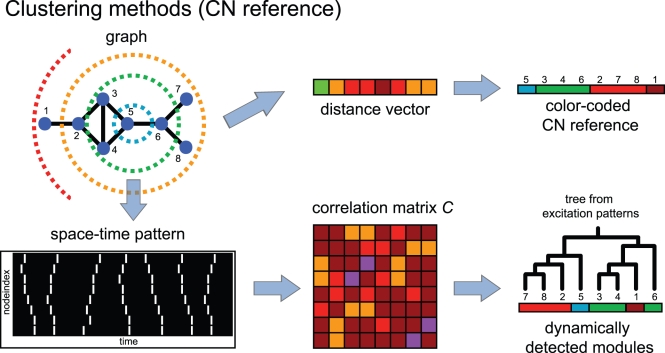
Construction of a color-coded topological reference which is based on the location of the CN in the network (top row), and computation of the dynamic clustering tree is carried out as described in Figure 2 (bottom row). (Top row) The central node *h* (inner circle in the graph representation) displays the highest betweenness centrality *B* (see Methods: betweenness). It is surrounded by modules of equidistant nodes (from *h*). The nodes of the resulting distance vector are re-sorted according to their distance to *h*.

In this study, we focus on two structural properties of networks and use them in terms of topological references. These properties are modularity and node centrality and they are represented by the topological modularity (TM) reference and the central-node based (CN) reference, respectively. To highlight the individual impact of each topological property on dynamic pattern formation we first probe different types of artificial networks dynamically and compare the results with the respective topological reference. We then validate our results with modified versions of these networks (see Figure S1 and Figure S2 in Text S1: Analysis of randomized network topologies) and with different types of hierarchically structured graphs, which represent the two topological properties to different extents. We finally transfer our analysis to more densely connected networks and to different hierarchically structured real-world topologies (see Methods for details on the construction of the respective references, the dynamic models and the different types of graph architectures and graph randomization processes). Figures 2 and 3 summarize our strategy of comparing the pattern of simultaneous excitations (correlation matrix *C*) with the corresponding topological feature, namely the topological modules (TM, Figure 2) and the central-node based reference (CN, Figure 3). Both, the graph and the simulated “space-time” pattern are converted into matrices giving the pairwise distances and the number of simultaneous excitations, respectively. The two matrices are processed further to yield the respective clustering trees, which then are sorted, color-coded and systematically compared (see Methods for a detailed description of this procedure.)

### Analysis of the Modular Topology

**Figure 4:**
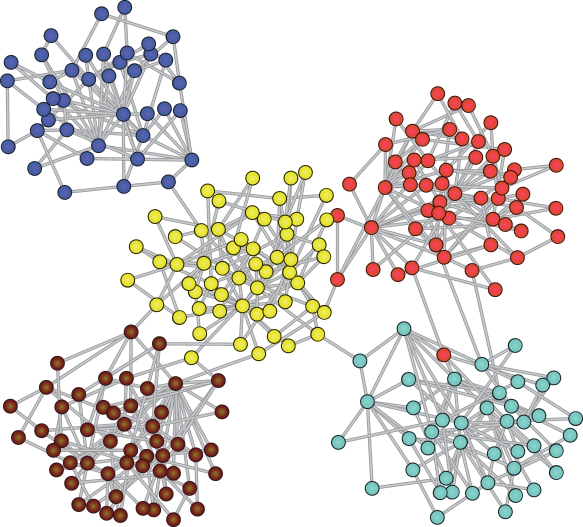
Graph representation of the modular scale-free network. The nodes are colored according to the dynamic clustering tree (resulting from a simulation with *f* = 0.01) after assigning a threshold for 5 modules (the number of topological modules). The dynamic clustering agrees with the topological modules almost completely.

We start our analysis with the modular scale-free network in order to test the explanatory power of the TM reference. As a first step we visualize for a single value of *f* how well the dynamically detected clusters follow the topological modules. We can map the clustering tree obtained from the correlation matrix onto the graph by thresholding it to yield the same number of modules *μ* as detected topologically and assign colors as labels to the modules. Figure 4 displays the corresponding graph with the modules colored exclusively on the basis of the dynamically detected clusters (DDCs), resulting from a simulation with *f* = 0.01. In this case, the dynamic clusters have a large overlap with the modules found topologically.

**Figure 5:**
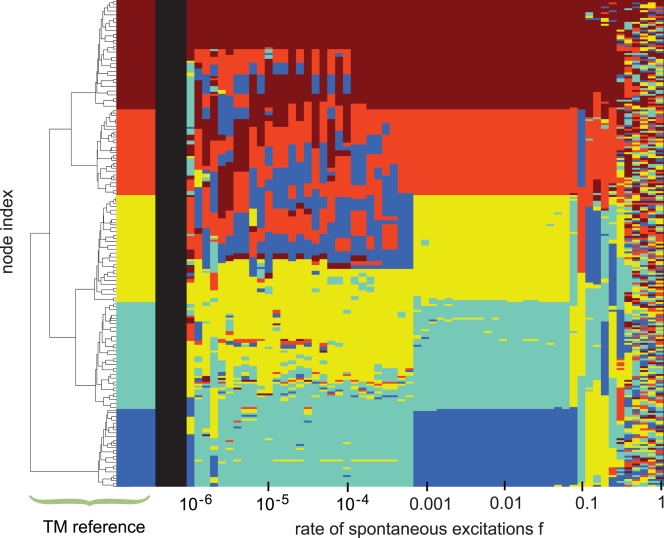
Dynamically detected cluster (DDC vectors) for 10^−6^<*f*<1 (right) re-sorted and colored according to the TM reference (left), as described in Figure 2. The region of the image displaying the highest consistency between the TM reference and the DDC vectors (10^−3^<*f*<0.1) marks the range, where the dynamics is able to exploit the given topological modules rather precisely. In this range of *f* the distribution patterns of excitations are dominated by burst regimes (as discussed in [36]. The pattern formation for *f*>0.1 is strongly influenced by random firing events, while for *f*<10^−3^ the modular boundaries are followed only partly by the dynamics, hinting at another form of correlation between dynamics and topology, which acts on a larger topological scale.

As a next step, we analyze the whole range of the parameter *f*. This is summarized in Figure 5. The color bar on the left-hand side represents the color-coded TM reference. The sequence of color bars from left to right are the color-coded DDC vectors for increasing values of *f*. There are three distinct ranges in *f* characterized by different patterns of the DDC vectors. Above a value of *f* = 0.1 any regularity is replaced by a random distribution of colors. Here, the random excitation events dominate the dynamics, thus leading to uncorrelated excitations and to a formation of unsystematic dynamically detected clusters. For lower values of *f* two different forms of node integration into dynamic clusters can be discriminated. Up to a value of *f* = 10^−3^ the DDC vectors are a mixture of homogeneous regions (representing well detected topological modules) on the one hand (in the bottom part of each DDC vector in this *f* range) and regions with smaller scale homogeneities on the other (top part of the DDC vectors). In this range the topological modules coincide partly with the dynamic clusters, but the dynamic integration fails to comply with the topological hierarchy of the modules. The middle range in *f* = 0.01 (10^−3^<*f*<0.1) is characterized by a very high order of the DDC vectors and an almost perfect agreement with the TM reference. Besides this systematic dynamic retrieval of the topological modules the DDC vectors in this *f* -range are also characterized by a strong consistency with the hierarchy of the modules on the level of the whole graph. The separation of the DDC vectors into two regimes with respect to *f* (omitting here the noise-driven high *f*-regime) is basically driven by the three-state model's behavior under spontaneous excitations. As pointed out in our previous work [36], the model displays a transition in the distribution patterns of excitations from a global (spike) to a more local (burst) regime with an increasing rate of spontaneous excitations *f*. While a spike (low-*f* regime) is able to reach most of the system (depending on the excess of nodes in the excitable state *S*), the burst (higher-*f* regime) is characterized by one or more excitation spots which propagate through the system on a localized level due to a more balanced distribution of the states *S* and *R* (Video S1 in Text S1 illustrates the propagation of excitations on a modular graph architecture during the burst regime). Consequently, the DDC vectors separate rather precisely at the position where the burst dynamics outbalances the spike dynamics. In this sense the burst dynamics provides a suitable tool for the dynamic retrieval of topological modules.

### Analysis of the Hub Dominance

**Figure 6:**
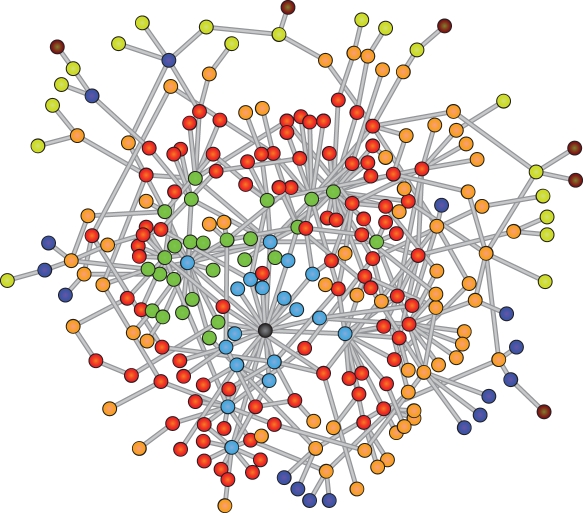
Network representation of the BA graph. The nodes are colored according to the dynamic cluster tree (resulting from a simulation with *f* = 10^−5^) after assigning a threshold for 7 modules (the maximal distance to the hub). Most of the dynamically detected clusters are arranged in a ring-like fashion around the central hub highlighted in black.

The results for *f*<10^−3^ suggest that another form of dynamic integration of nodes takes place beyond the module level. Groups of nodes which belong to different topological modules (see e.g. the blue and red labels in Figure 5) are placed in close dynamic proximity (that is, they are integrated into the same dynamic cluster). For testing this new principle of dynamic integration we repeat this simulation with a non-modular scale-free BA graph (see Methods) and the CN reference discussed in Figure 3. In Figure 6 the BA graph representation has been color-coded according to the dynamically detected clusters (with a preset value of 7 clusters, which determines the threshold applied to the corresponding clustering tree) at *f* = 10^−5^. One observes a rather clear ring-like arrangement of colors around a central node which is one of the hubs in the graph. This distribution of the dynamic clusters around a central node *h* (displayed in black) confirms our hypothesis that another topological feature is shaping the distribution of excitations in this low-*f* regime.

**Figure 7:**
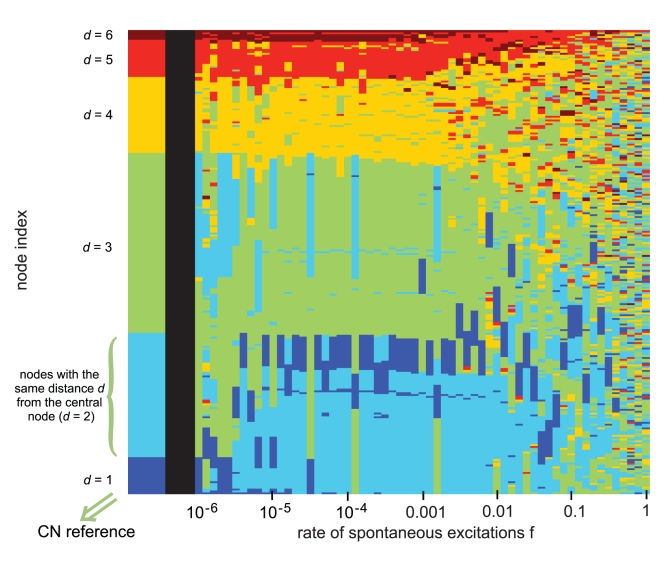
Dynamically detected clusters (DDC vectors) for 10^−6^<*f*<1 (right) re-sorted and colored according to the CN reference (left), as described in Figure 3. In this reference, the nodes sharing the same color have the same distance *d* to the central node *h* (see Figure 3 top row). Up to a value of *f* = 10^−3^, the equidistant nodes are almost completely integrated dynamically according to this topological reference. In this *f*-regime the dynamics is characterized by excitation waves (spikes), which cover the whole system and which emerge from *h* preferentially and independently of the location of the accidentally excited node. The increasing scattering of colors for higher values of *f* indicates a change of the dynamic regime, the spike dynamics is increasingly replaced by burst dynamics.

Studying the agreement between the CN reference and the DDC vectors for the BA graph over a whole range in *f* leads to the result shown in Figure 7. The CN reference (left-hand side) clusters all nodes *t* according to their distances *d* to the central node *h* with *d* = *L_ht_*. Up to a value of *f* = 10^−3^ all equidistant nodes assemble more or less in the same dynamic cluster and even the distance order is maintained (except for *d* = 1 and *d* = 2). Above *f* = 10^−3^ the homogeneity of the DDC vector drops rapidly finally reaching a random composition. Again this decrease of dynamic order is accompanied by a decrease of the spike regimes in the overall dynamics. The recurrent simultaneous excitations which lead to the observed pattern are caused by global properties of the graph's topology. We assume that such networks are able to channel the excitations produced by random events into their centers, which are composed of one or a few nodes displaying the highest betweenness centrality (as given by the number of shortest paths leading to the node; see Methods). From there, the excitation waves pass through the rest of the system reaching all equidistant nodes (seen from the center) at about the same time and thus integrate them dynamically. The dynamics in Video S2 in Text S1 contains several spike events which demonstrate the typical propagation of excitations in a BA graph. In addition Figure S5 illustrates the consistency between the sequential arrangement of ring-shaped modules (as seen from the central node) and the chronology of excitations showing the fraction of simultaneous excitations within each of these modules at the same time.

### Analysis of Hierarchical Network Topologies

An integration of both topological properties (modularity and hub dominance) into one system has been accomplished via the introduction of the hierarchical scale-free graph [10],[28]. We expect from the previous discussion that both levels of dynamic organization are present in such a network. As other network designs exhibit hierarchical properties as well, we contrasted different types of hierarchical graphs, also considering densely connected graph structures which, for instance, characterize many neuronal systems. To allow for the analysis of highly connected networks we extended our dynamic model with the additional node degree-dependent parameter *ω* (which regulates the excitability of a node, i.e. the number of excitations needed in order to trigger a firing event; see Methods for the exact definition of *ω*).

All hierarchical networks introduced here share a hierarchical fashion of linking the modules, but some of them lack the hubs and the scale-free degree distribution. One would expect that such graphs are not able to produce consistent ring-like excitation patterns as observed in the BA graph. In the following we will investigate how these topological properties determine the distribution pattern of excitations. We checked, however, that this general phenomenon does not depend on the exact method of generating a particular topological property.

We tested four different hierarchical networks, i) the hierarchical scale-free graph [10],[28], ii) a variant of the hierarchical scale-free model (which permits the construction of densely connected graphs), iii) the fractal modular network [23], and iv) the hierarchical cluster network [21]. We generated 10 networks of each graph type, simulated the dynamics, and computed *Q_dyn_* from the resulting dynamic clustering trees, as before. Densely connected networks were simulated with a threshold of *κ* = 0.1, as described in Methods.

**Figure 8:**
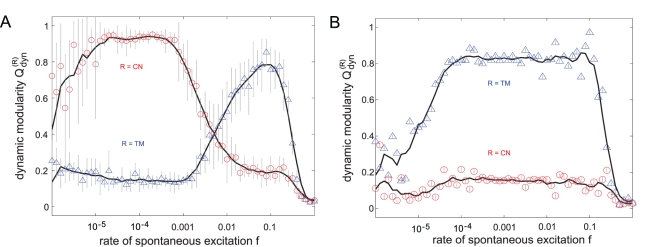
Levels of dynamic organization in different graphs with a hierarchical distribution of modules. The dynamic modularity *Q_dyn_* for both the TM reference (blue ▵) and the CN reference (red ○) is depicted as a function of the rate of spontaneous excitations *f*. (A) The hierarchical scale-free graph displays properties of both, the modular and the BA graph. Thus, the two levels of dynamic integration are visible within the same network for the respective values of *f*. The transition between these two levels corresponds to the transition from spike to burst dynamics. (B) The mapped fractal graph from [54] lacks a scale-free degree distribution and, consequently, hubs, which is reflected in low values of 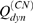. The absence of ring-like excitation patterns also explains the extension of the high-value range of 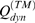 towards low values of *f*.

In the following the results are limited to the hierarchical scale-free graph [10] and the mapped fractal graph [23] as both other results agree well with their respective counterparts. Figure 8 displays 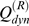 averaged over all networks as a function of *f* for the TM reference (blue ▵) and the CN reference (red ○). In the hierarchical graph (Figure 8A) the dynamic detection of the topological modules based on the TM reference works very well for high values of *f*. Increase and decline of 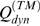 depend on the transition from spike dynamics to burst dynamics and on the increasing noise intensity *f*, respectively (Figure S3 displays the corresponding time series of the excitation density *ρ_F_* for three different values of *f*). This increase is accompanied by decreasing values of 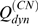 for the CN-dependent results which display their maximum in the low *f*-regime. Here, the high values of 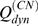 indicate a strong dominance of the hubs and their importance for the formation of the excitation waves. Indeed, this graph structure facilitates the emergence of both forms of dynamic organization. This observation, that certain types of hierarchical graphs can host both dynamic patterns with the rate of spontaneous excitations inducing a switch from one to the other, will be discussed in detail elsewhere.

In the mapped fractal graph (Figure 8B the absence of hubs prevents the generation of ring-like excitation patterns (as seen in the low values of 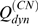) with the effect that the range of dynamically detected topological clusters (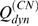) enlarges towards low values of *f*.

By an adjustment of the dynamic model the consistency to the more sparsely connected networks demonstrates that (i.e. by rescaling the excitability; see Methods) it is still possible to retrieve both dynamic regimes even in densely connected graph architectures, similarly to the more sparsely connected networks. Rescaling the excitability (by requiring more than one excitation in the neighborhood for exciting a node) thus provides a consistent extension of our original dynamics to higher connectivities.

### Analysis of Biological Neural Topology

Compared to metabolic reaction networks or protein-protein interaction networks, the architecture of many neuronal systems is characterized by a high density of connections [13],[39],[40]. We studied neuronal networks of two organisms at two fundamentally different levels of organization, namely the cortical systems network of the cat and the cellular neuronal network of the nematode *C. elegans*.

First, we analyzed the cortical network of the cat, which has a well-characterized topology [8],[13] and has been the basis of previous dynamical simulations [16],[41],[42]. We focused at connectivity at the systems level, which is more reliably established than cellular cortical connectivity. At the systems level, all the neurons of a cortical area are integrated into a single node. This coarse-graining approach scales the cortical network representation down to *n* = 55 nodes and 238 directed edges and 327 undirected edges which originate from 892 cortico-cortical connections.

Second, we considered the cellular neuronal connectivity of the nematode *C. elegans*, which has also been studied extensively. Due to the fixed number of nodes, the neuronal network of *C. elegans* serves as an excellent neuronal model system [43]. This version of the cellular neuronal network of *C. elegans* contained *n* = 277 nodes and 1731 directed edges and 187 undirected edges.

**Figure 9:**
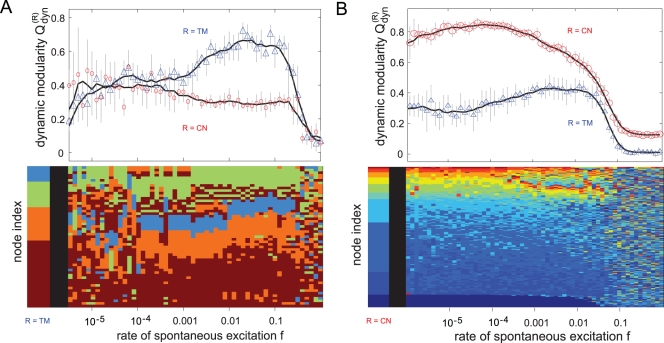
Levels of dynamic organization in two different neuronal networks. The highlighted curves (bigger symbols; top row) correspond to the respective DDC vector results (bottom row). (A) The dominance of modular elements in the cortical network of the cat is reflected by a distinct increase of *Q_dyn_* for the TM-dependent results in the high-*f* regime (blue ▵; top) as well as by the homogeneous clustering of the DDC vectors (TM-dependent results; bottom), while central node effects seem to play only a marginal role (see the slight superelevation in the low-*f* regime [red ○]; top). (B) By contrast, the cellular network of *C. elegans* displays a strong dependency on two adjoining central nodes which dominate the dynamics in a wide range of *f*. The drastic increase of the CN-dependent results for *Q_dyn_* in the low-*f* region (red ○; top) reflects the high order of the DDC vectors (CN-dependent results; bottom) with a conserved distance ranking of the topologically detected node clusters. Even here, there exists a noticeable but comparatively subordinate influence of the module-based excitation patterns (blue ▵; top).

The connection density of the cat cortex representation is comparatively high (*z* = 0.3), while the connection density of the neuronal network of *C. elegans* is about tenfold smaller (*z* = 0.028). Therefore, we decided to use the modified DE model for the cat cortex with *κ* = 0.15 and *p* = 0.1 and the original DE model for *C. elegans* with *p* = 0.01. We analyzed both networks in the range of 10^−6^<*f*<1. The TM references consist of 4 modules (cat) and 8 modules (*C. elegans*), respectively. The four modules in the cat systems network correspond to those previously identified by other clustering approaches [13], and represent sets of visual, auditory, sensory-motor and fronto-limbic cortical areas. The diagrams (Figure 9 top) display the analysis of the dynamic modularity 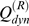 for both topological references. The diagrams on the bottom show corresponding curves with highlighted markers on the top. They display the TM-dependent DDC vectors for the Cat (Figure 9A bottom) and the CN-dependent DDC vectors for *C. elegans* (Figure 9B bottom).

Examining the relation between topology and dynamic properties independently of the organism, both networks show certain characteristics of a hierarchical scale-free network [10],[28], that is, the typical differences in the dynamic dominance of modular and hub features for different levels of spontaneous activation (as indicated in the *f*-dependent course of *Q_dyn_* in Figure 9 top), which implicate the existence of a complex hierarchical structure. However, both organisms also exhibit great differences in their dynamic regimes.

For low levels of spontaneous excitation in the cat cerebral network (Figure 9A top), the CN and TM references are equally well related to the network's dynamic behavior. The strong correlation between dynamics and the modular topology is reflected in a high consistency between the TM reference and DDC vectors in the high *f*-regime (Figure 9A bottom) also indicated in Figure 9A top in 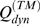, while there seems to be only a marginal influence of hubs. If we exchange the TM reference by the modules previously identified for the cat cortical network [13], the general features of *Q_dyn_*(*f*) remain intact (in particular the clear peak in *f*; see Figure S4).

On the other hand, the dynamic behavior of the cellular network of *C. elegans* is for all but the highest levels of activation dominated by the distance to a central node (Figure 9B). Betweenness analysis revealed two nodes in direct neighborhood, which display the highest node degrees of the neuronal network, and which may serve as an initial point of circular excitation waves. Nodes 52 (AVAL) and 53 (AVAR) display the highest node betweenness (and the highest node degrees). The distance between both nodes is 1, as they are mirror-symmetric versions of the same neuron, AVA, on the L and R sides of the nematode's body.

## Discussion

### Overview

The current paper presents some aspects of a pattern-based computational approach for linking network topology and dynamics. This approach proved useful in probing the functional organization of complex biological networks. The comparison of topological features and simulated network dynamics demonstrated that features such as central hub nodes and network modularity can strongly and systematically shape a network's dynamic behavior. Moreover, in hierarchical modular networks, where multiple of these features were present, the network dynamics exhibit a functional switch for different levels of spontaneous network activation between the dynamic organization through a central node or through modular features.

The method also reveals the dynamic impact of different topological characteristics in biological neural networks. In particular, the dynamics in the cellular neuronal network of *C. elegans* appears organized by the topological distance to a central hub node, whereas the dynamic behavior of the cat cerebral cortical network appears more strongly influenced by network modularity. Both topological features, however, contribute to the organization of the networks synchronization dynamics. Given the restricted size of the biological networks, the functional implications of the features would have been difficult to derive from a conventional analysis of the networks' degree distributions. These findings have implications for understanding the relationship of network topology and dynamics in complex neural networks, as detailed in the following sections.

### Model Limitations

The presented approach draws on a simple dynamic model for describing excitable elements. This model only represents node activation, inactivation, as well as a refractory period, with discrete time steps. Given the complex dynamic behavior of neurons and neuronal systems, the model may appear overly simplistic. However, we believe that the model captures essential features of excitable elements, such as the principal activation cycle of neurons. Moreover, at the moment it is far from clear how much detail is required to realistically describe the interaction of excitable elements in networks. A good starting point for analyzing such pattern-formation aspects also in more sophisticated models could be built upon the parallel to a recent simulation study of the cat cortical network, which uses a more sophisticated population oscillator model to describe the activity of individual cells within the cortical areas [16]. This study led to a similar finding of a modular dynamic organization that strongly followed the modular topological organization. There are also precedents for the successful application of highly simplified models of cortical networks. For example [41] used a simple spreading model to infer basic properties of the relationship between node lesions and network activity in the thalamo-cortical network of the cat. Similarly, [42] replicated epileptiform steady-state activation patterns in the cat cortical network with the help of a simple thresholded spreading model. In addition, in the present work the model parameters were varied over a wide range; however, the different simulations resulted in similar principal behavior.

### Topology and Dynamics of Neural Systems

When applied to biological neural networks, our approach revealed that the dynamic behavior of neural networks may be coordinated via different topological features. While activity in the neuronal network of *C. elegans* is shaped by excitation spreading from central hub nodes, the dynamic behavior of the cat cortical network is largely dominated by the network's modular organization. Moreover, the cortical network may switch from modular to hub dominance for low levels of spontaneous activation.

The current analysis applies to network dynamics with spontaneous node activations, as observed in tonic neural activity, but without explicit external (sensory) input. This description corresponds to the experimental case of so-called resting state connectivity, a type of functional connectivity that persists in the absence of specific external stimulation. Resting state networks have been studied intensively over the last years and have been considered as default frameworks of neural dynamics [44]. Resting state connectivity can be derived experimentally from time-series correlations between large-scale brain regions, such as cortical areas. The regions' activity is estimated from different functional imaging techniques (e.g., EEG, fMRI); and typically, the coupling occurs at very low frequencies, around or below 0.1 Hz [45]. The slow-frequency coupling may be a reflection of faster electrophysiological coupling among distributed neuronal populations [17]. Experimental resting state data are currently available for cortical networks in humans and non-human primates, but not for the cat cortical network studied here. However, the present theoretical findings largely agree with what is known from the available experimental data. For instance, resting state data for human and primate cortical networks at the systems level show a strongly modular organization [46],[47]. Earlier experimental findings, based on activity spreading after local cortical disinhibition, also suggest that primate cortical areas co-activate, in groups that closely match the known topological clusters [15]. In addition, previous theoretical studies also support the conclusion that the dynamic organization of large-scale cortical networks in the absence of external stimuli is strongly shaped by the networks' modular structural connectivity [16].

However, it was also suggested that hub-like areas exist in cortical networks which possess a relatively large number of connections and which can be identified implicitly from the networks' behavior after simulated node lesions [8],[24],[48]. The leading central nodes identified here for the cat cortical network by node betweenness, multimodal areas 35 and AES, are also among those suggested previously by degree and lesion impact [8],[24]. For low rates of spontaneous activation, the cortical dynamics became somewhat more strongly correlated to hub distance than network modules. This dynamic switch characterizes the cortical connectivity as a complex hierarchical network and indicates the possibility that particular cat cortical areas act as hub-like nodes for the organization of low-noise dynamic regimes. This point still needs to be investigated in more detail. Importantly, only coarse large-scale activations can be resolved with the current neuroimaging techniques. Nonetheless, it is clear that cortical networks have a multi-level modular organization (forming clusters of sub-clusters of excitable nodes [21], with modules spanning from cellular cortical circuits and columns to clusters of strongly interlinked areas). Therefore, it can be speculated that, once data for additional scales of cortical networks are available, switches of the dynamic behavior between different topological features become more clearly apparent.

In contrast to the cortical network the dynamic behavior of the *C. elegans* network was dominated by central node distance for all levels of spontaneous activation. Experimental findings also indicate that neuronal dynamics in *C. elegans* are coordinated by central pattern organizers [49],[50] rather than through network modules. Indeed, the pair of AVA neurons, which have the highest degree and highest node betweenness in the *C. elegans* network, and which therefore may be considered as network hubs, have been implicated as a component in a central pattern generator responsible for locomotion control [49]. Specifically, AVA is thought to be responsible for backward movements. The present results suggest that this node may also have a more general function in coordinating dynamic activity in the nematode nervous system.

The finding of dynamic organization through network modules in large-scale cortical networks, versus organization through few central nodes in cellular neuronal networks, makes intuitive sense. Given the small size of its nervous system, the functional specialization in *C. elegans* occurs at the level of individual cells, which exert their roles globally across the network. On the other hand, specialization in the mammalian cortex arises for whole brain regions (e.g., visual cortex, sensory-motor cortex) comprising several cortical areas which are closely cooperating within modules to perform the various aspects of their functional subdivision.

### Conclusions and Outlook

When studying dynamics on networks, the synchronization behavior of each single node is a suitable indicator to estimate the dynamic scope provided by a graph's topology. Different forms of synchronization require different structural properties. By the application of a simple excitable medium (the DE model) we were able to generate two distinct forms of synchronization via the regulation of a single dynamic parameter, the amount of spontaneous excitations *f*. This noise level *f* also defines the (length) scale on which a specific dynamic process will predominantly be situated. Consequently the (larger-scale) wave-like propagation (consistency with CN reference) is dominant at lower levels of *f*, while the local module-based synchronization (consistency with the TM reference) is situated preferentially at higher *f*.

Via comparison to two different topological references representing the elementary graph properties modularity and hub dominance the dynamic results were attributed to the respective synchronization behavior. In the burst range of *f*, networks exclusively featuring modular properties with decentralized hubs display synchronization behavior predominantly within their communities as indicated by the consistency to a module-based topological reference. If a graph is dominated by one or a few hubs in its center (a feature of the BA graph) a global (ring-like) synchronization phenomenon is visible due to the formation of excitation waves which reach the whole system from the graph's center. In contrast to our modularity definition it is more difficult to decide whether a node is the center of a graph or not. Here, we used the betweenness centrality (*B*) definition, but the results indicate that *B* does not alone account for the unifying topological quantity for different networks. The analysis of different hub categories [10],[11],[12] and their involvement in organizing the dynamics [24] is an important next step of the study described here. We did not do this so far, because it would require simulating substantially larger networks to obtain reliable results. We would also like to point out that the prototypes of pattern formation we identify, might serve as minimal models of the brain activity regimes reported by Izhikevich and Edelman in their model of mammalian thalamocortical systems, which emerge spontaneously as a result of interactions between architectural features and the dynamics [51]. An important challenge for the future will be to activate modeled neural networks more selectively with patterns representing functional inputs, and to observe the interactions of stimulus-related activity with default activity.

In summary, by using a simple dynamic model we could determine a “network equivalent” of pattern formation, where patterns are represented by correlations between topology and dynamics. Specific topological features give rise to and regulate quantitatively certain elementary forms of patterns. We believe that this correspondence is not restricted to the specific dynamics considered here. The recent findings on synchronization of phase oscillators [52],[53] show similar matches between topology and dynamics as the results reported for an excitable system. In this light a comparison of these systems in detail (our discrete excitable three-state model and the continuous phase oscillator model) would be very interesting and could point towards common links between topology and dynamics far beyond individual dynamical systems. It is particularly interesting that the authors employ phase oscillators and their synchronization properties also to determine functional groups in the neural system of *C. elegans* [54].

## Methods

### Simulated Network Topologies and Network Modification

#### Overview

This work is based on a variety of network architectures, topological parameters, and dynamic techniques. The basic artificial network types and methods presented in the first part best suit our objective to rule out the individual impact of modularity and hub dominance on dynamic pattern formation. The second part deals with hierarchically structured networks and with real-world topologies, that is, biological neural networks, which will be analyzed concerning both topological properties. The third part contains the analysis tools to probe the networks topologically and dynamically.

#### Scale-free network

This basic network type is constructed via preferential attachment following the Barabási-Albert (BA) model [1]. The generation of this network starts with a small set (we use *n*_0_ = 2) of completely connected nodes. Then, new nodes are added to the graph and connected with *m_A_* edges (we use *m_A_* = 1.25) preferentially to the nodes with the highest degrees (details on the BA algorithm for non-integer values of *m_A_* are given in [36]). A typical network of this type is shown in Figure 4. It consists of *n* = 250 nodes, *m* = 313 edges, and a connectivity of *z* = 0.01 (with *z* = 2*m*/(*n* ^2^−*n*)). The nodes in this network are hierarchically distributed in the following sense: during the growth process the hubs are more likely connected to each other than to other nodes, thus forming the center of the graph, while the nodes with small degrees contribute to the periphery for the most part.

#### Scale-free modular network

This network type consists of several modules of approximate identical size. We used a modification of the community model [4],[55] to generate graphs with 5 modules (*n* = 250, *m* = 496, and *z* = 0.016 [*n* = 250, *m*≈515, and *z*≈0.0165 for the analysis of the randomized topologies]). According to the BA graph generation rule, each module starts with a small number of fully connected nodes (*n*_0_ = 2). All further nodes are attached preferentially with *m_A_* = 2 edges until the average size of each module is reached. At last, each module pair is connected with *m_E_* = 1 (*m_E_* = 3 for the analysis of the randomized topologies) random edge on the average. In contrast to the BA graph, the hubs are distributed among the modules.

#### Randomized network topologies

We use a systematic randomization process to modify an existing network topology in a directed way. In this procedure two linked pairs of nodes are randomly selected and rewired (i.e. the two edges are reassigned among the four nodes) as long as neither network fragmentation occurs nor double or self-edges form. In the course of the first variant of this randomization procedure (process 1), the topological modularity *Q_top_* (determined as described, e.g., [4],[56]) of a graph is reduced by randomly selecting pairs of nodes in different modules and cross-linking them, thus increasing the amount of inter-modular edges. To avoid the formation of a hierarchical structure we ensured cross-linking between nodes with a degree *k_s_*<*median*(*k_N_*) with *N* = (1,2,3,‥,*n*). During the other variant of the randomization procedure (process 2), the influence of the hubs, specified by the betweenness centrality *B*, is reduced by first selecting an edge connecting two nodes with *B*>0.4·max(*B_N_*) and then selecting a second edge with *B*>0.2·max(*B_N_*). The BA graph exhibits only a small amount of nodes with betweenness values above this threshold. The elimination of the most important edges ensures a drastic degradation of the central hub significance with increasing randomization depth.

#### Hierarchical scale-free network

Hierarchical scale-free graphs [10],[28] have been introduced to account for both, hub dominance and modular clustering. The graph generation is based on a fractal algorithm which uses multiplication and cross-linking of existing graph structures to produce a deterministic scale-free network with self-similar elements. Compared to a BA graph, the degree *k_h_* of the central node *h* of the hierarchical graph is notedly high (*k_h_* = (−3+3*^it^* ^+1^)/2 with *it* denoting the number of iterations in the generation rule). Such a network would still display an unbalance in both levels of dynamic organizations. To reduce *k_h_* we added the probability *g* for an edge to form between *h* and the respective other node during the generation process. Starting with a set of 4 completely connected nodes and applying the rules in [2] one would yield a graph with *n* = 256 nodes and *m* = 780 edges after 4 iterative steps. For *g* = 0.5 the resulting networks possessed *m*≈650 edges.

#### Fractal modular network

The fractal modular network displays some basic properties of the hierarchical scale-free network, but its fractal connection scheme disagrees completely. This network has been introduced by Sporns et al. [23] for the analysis of the cerebral cortex which is also characterized by multiple hierarchical levels. We constructed a mapped fractal graph with six hierarchical levels according to the following parameter constellation. We preset parameter *E_s_* which in principle regulates the connectivity of the graph to a value of *E_s_* = 2. Starting with a complete graph of 8 nodes (*m_S_* = 3 and *n_S_* = 8) the resulting graph comprises *n* = 256 nodes and *m* = 3456 edges (for details on the generation of the mapped fractal networks see Sporns et al. 2006 [23]; the index *S* denotes the variables which are used in [23]).

### Biological Neural Network Data

We applied the analysis approach to two sets of neural network data at different scales of organization. The first data set describes systems level connections between different areas of the cat cerebral cortex, and is based on a global collation of cat cortical connectivity (892 interconnections of 55 areas). This collation was developed from the data set described in Scannell et al. (1995) [57] and forms part of a larger database of thalamo-cortical connectivity of the cat [39]. The database was created by the interpretation of a large number of reports of tract-tracing experiments from the anatomical literature.

The second data set represents cellular neuronal connectivity of the nematode *C. elegans* (277 neurons and 2,105 synaptic connections). This data set was adapted from Achacoso and Yamamoto (1992) [43]. That compilation is largely based on the dataset of White et al. [58] in which connections were identified by electron microscope reconstructions. The previously presented connectivity data [43] was modified in the following way. Neurons of the pharyngeal ring, for which there was no internal connection information, were removed from the network, leaving 280 neurons. In addition, three neurons (AIBL, AIYL, and SMDVL) were removed, because of lacking spatial information. Eventually 277 neurons were included in the analysis. The size of the global and local *C. elegans* datasets analyzed here was comparable to that used in previous studies. For example, studies of the small-world properties [37] or characteristic motifs [12] of *C. elegans* considered 282 and 187 neurons, respectively. Both chemical and electric synapses (gap junctions) were included as connections in the analysis.

### Topological References

In order to understand how topological properties and dynamic observations are related, we will address our quantification schemes for topology and dynamics separately at first.

We determine two topological references which are both based on the pairwise distances of all nodes within a network. Let the distance *L_st_* be the shortest path connecting node *s* with node *t* The first reference is based on the topological modules (topological module reference, TM, see Figure 2 top). It is computed from the distance matrix *L* = *L_st_* which is then analyzed with a standard hierarchical clustering method. We tested single-linkage, complete-linkage and average-linkage approaches and found basically no differences between these methods for the task at hand. In the following, we used UPGMA (Unweighted Pair Group Method with Arithmetic mean) clustering, that is, the pair-wise combination of nodes or groups of nodes with minimal distance which is determined by the arithmetic means of the respective groups. The relative positions of the nodes which are the leaves of the topological reference tree obtained in this fashion are a condensed representation of all distance relations within the network. A similar way of analyzing the module structure uses the topological overlap [14]. The modules predicted with this method can be recovered from the topological reference tree by horizontally cutting the tree at a certain hight. The tree fragments resulting from this thresholding procedure serve as module predictions. In principle one has to analyze the dependence of the module predictions on threshold variation or conversely one can determine the threshold by prescribing the number of modules *μ*. Assigning a label (e.g. a color) to each node within a particular module leads to the final result, the TM reference, for which agreement with the distribution patterns of excitations can be checked.

The second topological reference is based on the central node *h* of the network (central node reference, CN, see Figure 3 top). Although many properties can in principle contribute to the centrality of a node, we will here select node *h* to be the one displaying the highest node betweenness *B* [59]–[61]. The distances between *h* and all other nodes form a distance vector. All nodes with the same entry in the distance vector (e.g. equidistant nodes from *h*) are taken to form a cluster, representing this topological reference (CN clusters). Resorting the distance vector accordingly yields the color-coded CN reference. Here, the number of clusters *μ* is given by the maximal distance from node *h*.

### Dynamic Models

Dynamics were simulated on the graph architectures using the discrete excitable (DE) model described in the introduction. We used 35000 update steps (first 10000 updates were discarded) with the following parameter constellation: the rate of spontaneous excitations *f* was varied in the range of 10^−6^<*f*<1 to systematically study the impact of noise on the formation of the excitation patterns; recovery probability *p* was set to a constant value of *p* = 0.1; the initial condition was a random equipartition of the states *E* and *T*. This parameter constellation will be used in all of the studies presented here.

In the basic DE model highly connected networks are in principle characterized by burst dynamics. Indeed, spikes emerge at very low values of *f* even here, but with a sufficiently high simulation time they are outbalanced by burst dynamics. We solved this problem by introducing parameter *ω* in our excitable model system. This threshold depends on the degree *k_s_* of a node *S* and determines the number of excitations necessary to turn a susceptible node into the excited state. In this variant all incoming excitations are stored in node *S* until *ω_s_* = *k_s_*·*κ* (with a minimum value of *ω* = 1) is reached.

### Comparison between Topological and Dynamic Organization

In order to allow for a direct comparison with topology, we base our analysis of the dynamics on pairwise node comparisons: for each pair of nodes we count the number of simultaneous excitations *σ_st_* in the given time interval. Properly normalizing these quantities to arrange between 0 and 1 (*σ* ˜*_st_*) and converting the corresponding matrix into a distance matrix *C* = *C_st_* = 1−*σ* ˜*_st_* leads to the correlation matrix *C* which represents the distribution patterns of excitations for a given graph and a given parameter constellation of the DE model. We aimed at understanding to what extent a selected topological reference is capable of explaining the patterns in the correlation matrix. To this end, the matrix can now be converted into a clustering tree (again by using UPGMA: see Topological references). The idea is now to rearrange the branches in the tree to best fit a given reference vector. The corresponding sequence of nodes constitutes the final result for the dynamics, namely the vector of dynamically detected clusters (DDC vector). The reference of the sorting vector can be any of the two topological references discussed above. Figures 2 and 3 summarize our analysis strategy. For the sorting we use an alignment algorithm which switches two neighboring branches at any position in the tree (obtained from the excitation patterns) as long as the similarity to the topological reference is increased. The decisive factor concerning the comparison of a pair of branches is the individual module composition of the respective leaves indicated by the mixture of (color) labels. A similar technique for the comparison of clustering trees has been introduced in [62].

### A Measure of Dynamic Modularity

For computation of our new quantity assessing the match between topology and dynamics, the dynamic modularity *Q_dyn_*, we compare two clustering trees, one coming from topology (with the clusters in the tree matching the modules in the graph), the other coming from the dynamics (more specifically: the matrix of simultaneous excitations). Cutting the first tree at a certain height (given by the module number, which is a parameter in our analysis) yields a set of modules, which we label by colors. Copying these node labels in the topological tree to the dynamic tree, and sorting for as many matching colors as the tree structure allows, permits us to quantify the color matches and mismatches between the two trees. Our null model is randomly distributing color labels on the graph (i.e. a sorting task of the dynamics tree to a random topological reference). As all these quantities depend strongly on the numbers of nodes in each module (or reference class), we normalize them to these sizes. In practice, this normalization is only important when we have very different sizes of modules in a graph. In this way we can assess whether the matching between a topological feature (here: the modules) and the dynamics (represented by the matrix of simultaneous excitations) is higher (or, in principle, even lower) than expected at random.

The same holds for the other topological reference, the CN reference, where the labels are provided not by a clustering tree, but by the distance from the central node. The possible values of 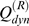 for a topological reference *R* lie between zero and unity with 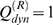 indicating the strongest agreement to the topological reference. Values below unity hint at a deviating distribution of nodes in the dynamic cluster tree.
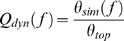
For both the topological reference and each DDC vector the distribution values *θ* are determined via comparison of the scattering of nodes *π* belonging to the same topological module *i* (as indicated by the color) with a null-hypothesis of this color distribution which is the average standard deviation (in *l* = 1,000 realizations) of the same amount of nodes randomly scattered over the whole network size *n*. The resulting quotient is normalized to the size of each module *n_mod_*.
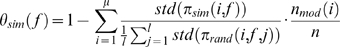


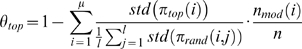



## Supporting Information

Figure S1 Computation of the average dynamic modularity <*Q_dyn_*> as a function of the topological modularity *Q_top_* for different network realizations of the modular scale-free graph. Depicted are the TM results (blue △) which have been averaged over the range of 0.01<*f*<0.1 and the CN results (red ○), averaged in the respective range of 10^−6^<*f*<10^−5^. The modular graphs (*n* = 250, *m*≈515 with *m_E_* = 3) were randomized in several steps producing networks with similar graph statistics but a decreased modularity. (A) Average randomization path of 10 different randomizations of the same network. The strong correlation between the TM dependent values of <*Q_dyn_*> and the topological modularity *Q_top_* proves the assumption that this level of dynamic organization has to be regarded as a consequence of the particular exploitation of modular network structures via burst dynamics. The respective exploitation via spike dynamics remains small and comparatively constant. (B) A similar behavior is also true for different network realizations and their respective randomization paths. These networks display the same correlation between <*Q_dyn_*> and *Q_top_*. One randomization path from (A) has been highlighted.
(0.87 MB EPS)

Figure S2 Computation of the average dynamic modularity <*Q_dyn_*> as a function of the hub dominance *B̃* for different network realizations of the BA graph. Corresponding to Figure S1A and Figure S1B, different BA graphs and their randomized versions have been examined. (A) The randomization procedure causes a decrease of the hub dominance and, accordingly, a reduction of the CN-dependent values of <*Q_dyn_*>. These results confirm the assumption that the whole graph structure and the central node in particular are responsible for the emergence of ring-shaped excitation waves, whose regularity is more and more disturbed with increasing randomization steps. (B) The randomization versions of the different networks are separated across the decreasing curve, but show nevertheless the same correlation as in (A).
(0.89 MB EPS)

Figure S3 Sections of time series of the excitation density *ρ_F_* of the hierarchical scale-free graph (see Methods) for different rates of spontaneous excitations *f*. (From top to bottom) Increasing parameter *f* induces a change of the dynamic behavior from spike dynamics to burst dynamics with a transition region of *f* displaying a mixture of both dynamic regimes.
(0.74 MB EPS)

Figure S4 Dynamic organization within the modular structure of the cortical network of the cat for two different definitions of the individual module composition. The curve indicated by the blue triangles corresponds to the TM-dependent results obtained from a UPGMA cluster analysis of the graph's distance information (using a threshold for 4 modules; see also Figure 9A). Similar results (green curve; errors are of the size of the other results) were obtained from simulations using a different TM-reference consisting of 5 modules which have been identified in a work of Hilgetag et al. (2000). The additional module contains three nodes which could not be assigned to the remaining modules. Concerning the individual composition, both references display a high consistency (75%).
(0.74 MB EPS)

Figure S5 Average fraction of excited nodes within each module resulting from previous excitations in a simulation of the scale-free (BA) graph in the spike-regime (*p* = 0.1 and *f* = 10^−4^). In the presence of excitations within the BA graph at a given time *t* the respective module (which is the concentric arrangement of nodes resulting from the CN-reference) with the strongest excitation density *ρ_F_*, i.e. the biggest fraction of excited nodes compared to its module size, has been identified. As a function of this module (the numbers on the abscissa denote the distance of the modules to the central node) the distribution of excitations over all modules has been computed for the following time step t+1 and depicted on the ordinate as the module-specific fraction of excited nodes. Based on the central node and the resultant concentric modules there is an apparent propagation of the excitations in the spike-regime from the center of the graph to its periphery including an average module-specific excitation of 45 to 65 percent of the respective nodes.
(0.67 MB EPS)

Text S1 Supporting Information
(1.30 MB ZIP)
