# Supplementary material for: Organization of Excitable Dynamics in Hierarchical Biological Networks
Source: PLoS Comput Biol. 2008 Sep 26;4(9):e1000190. doi: 10.1371/journal.pcbi.1000190 (PMC2542420; doi:10.1371/journal.pcbi.1000190)
Supplement: Text S1 — Supporting Information (1.30 MB ZIP) [file pcbi.1000190.s006.zip › Supporting_Info_1.pdf]

By means of the two topological references (TM-based and CN-based) we are able to characterize two elementary forms of propagating excitations influenced by both the rate of spontaneous excitations and by particular topological properties of the graph. In order to study the relation between the dynamics and the discussed topological properties more systematically, we now modify both types of graphs in a way, which successively reduces the modularity (for the modular graphs) and the importance of the hubs (for the BA graphs) keeping the other respective property approximately constant. According to our hypothesis that distribution patterns of excitations are, in fact, determined by the respective topological property, these graph restructurings should have an immediate and systematic impact on the dynamic organization of excitations. We used an edge-switching algorithm for both cases which conserves size n, connectivity $z = 2m/(n(n-1))$ (with the number of links m) and the sequence of individual node degrees k of the network during the restructuring process. In order to quantify the dynamic clustering in the respective regimes of f we introduced a new measure for the agreement between the dynamic clusters and the selected topological reference, the dynamic modularity $Q^{(R)}_{dyn}$ for a topological reference R (see the Methods for a detailed definition of $Q^{(R)}_{dyn}$ and the randomization procedures).

For the analysis of the modular networks we generated 10 different graphs (number of nodes $n = 250$, number of links $m \approx 515$ with at least $m_E = 3$ links between the modules) each with 5 modules. We performed the randomization algorithm (process 1; see Methods) in steps of 5, 10, 15, ..., 40, 50, 60, and 75 switches and determined the TM reference, the CN reference, and the dynamic clustering trees for each graph. For each of the randomization procedures we then compute the dynamic modularities $Q^{(TM)}_{dyn}$ and $Q^{(CN)}_{dyn}$, which quantitatively describe agreement between the dynamic clustering tree and the respective topological reference. More precisely, for the TM reference we computed the average dynamic modularity $\left\langle Q^{(TM)}_{dyn} \right\rangle$ in the range of $0.01 < f < 0.1$ and for the CN reference we computed $\left\langle Q^{(CN)}_{dyn} \right\rangle$ in the range of $10^{-6} < f < 10^{-5}$. Fig. S 1 shows $\left\langle Q^{(R)}_{dyn} \right\rangle$ as a function of the topological modularity Q_{top} for both the TM reference (blue Δ) and the CN reference (red ○). Fig. S 1A displays the average values over 10 randomizations for the single network. With increasing randomization steps the topological modularity Q_{top} exhibits a steady reduction which is accompanied by a drastic decline of the TM-dependent dynamic modularity $\left\langle Q^{(TM)}_{dyn} \right\rangle$, while

the CN-dependent results remain comparatively constant.

From the dynamic perspective two effects are visible in the relevant ranges of f. First of all, the systematic exploitation of the modules via localized excitation patterns (bursts) becomes more and more difficult, as the module boundaries disintegrate, causing the distinct decline of the respective values of $Q_{dyn}^{(TM)}$. Secondly, no ring-like excitation patterns emerge in the low-f regime even after 75 randomization steps, because the hubs are still distributed among the modules (simulation data behind Fig. S 1 not shown).

By providing results for different network realization and their randomization paths, Fig. S 1B confirms the general systematics, as all networks tend to group on the descending curve (TM). For each reference, a single randomization path for the network in Fig. S 1A has been exemplarily highlighted. These networks still display a comparatively high value of $\left\langle Q_{dyn}^{(CN)} \right\rangle$. This effect results from a module-based form of hierarchy, which is apparent in networks with a small number of inter-modular edges. Here, the excitations reach a module via a single or a few edges, respectively, consistently producing the same ring-like excitation pattern within the module.

We repeated this investigation with the BA graphs ($n = 250$, $m \approx 625$ with the average number of links in the iteration process $m_A = 2.5$) and the respective randomization procedure (process 2; see Methods). Here, we performed the algorithm stepwise (10 steps altogether), recalculating the betweenness centrality B after each iteration and using each resulting graph to determine the CN reference, the TM reference, and the dynamic clustering trees. We computed $\left\langle Q_{dyn}^{(R)} \right\rangle$ as above using the same parameter ranges in f. Fig. S 2 shows the dynamic modularity $\left\langle Q_{dyn}^{(R)} \right\rangle$ as a function of the hub dominance $\widetilde{B}$. Note that here we pass from the betweenness centrality B to the related hub dominance $\widetilde{B}$ in order to achieve a higher topological comparability of different randomization runs. The hub dominance $\widetilde{B}$ for a node v is computed with $\widetilde{B}(v) = \sum_{s \neq v \neq t \in V} \sigma_{st}(v)$ with $V \in \{1, 2, 3, ..., n\}$ and $\sigma \in \{0, 1\}$. If there is at least one shortest path for a combination of s and t which touches node v then $\sigma_{st}(v) = 1$. Fig. S 2A depicts the result for a single network. The dynamic modularity $\left\langle Q_{dyn}^{(CN)} \right\rangle$ displays a strong decrease (with increasing randomization, i.e. with decreasing hub dominance) already after a few iterations, while the respective TM results remain constant at a low level. The strong correlation between $\left\langle Q_{dyn}^{(CN)} \right\rangle$ and $\widetilde{B}$ supports the idea that in this parameter regime ring-like patterns are essentially determined

by the topological concept of a central node and, furthermore, that quantities like the betweenness centrality or the hub dominance define the central node. The separation of the hubs via this randomization process entails a systematic destruction of the hierarchical structure of the BA graph. The emergence of clearly separated hubs gives rise to a strong interference of the various excitation patterns and it may cause the excitation waves to pass unsystematically through the system depending on the location of an accidentally excited node. The results for different graphs and their randomization paths (Fig. S 2B) display a large consistency with the single-graph result. However, the randomization paths are distributed across the decreasing curve as indicated by the single highlighted result (black curve), as different graph randomizations populate slightly different regimes in $\widetilde{B}$.

Movie captions

Movie S1:
Section from a time series (from a simulation with $p = 0.1$ and $f = 10^{-3}$) which shows the excited state E mapped on the architecture of a modular graph. The continous excitations within the burst-regime, which propagate through the whole graph, accumulate particularly within the topological modules.

Movie S2:
Section from a time series (from a simulation with $p = 0.1$ and $f = 10^{-4}$) which shows the excited state E mapped on the architecture of a scale-free (BA) graph (corresponding to Fig. 6). Independent of the initial point of an spontaneous excitation the dynamics displays ring-like excitation waves within the spike events.

Figures

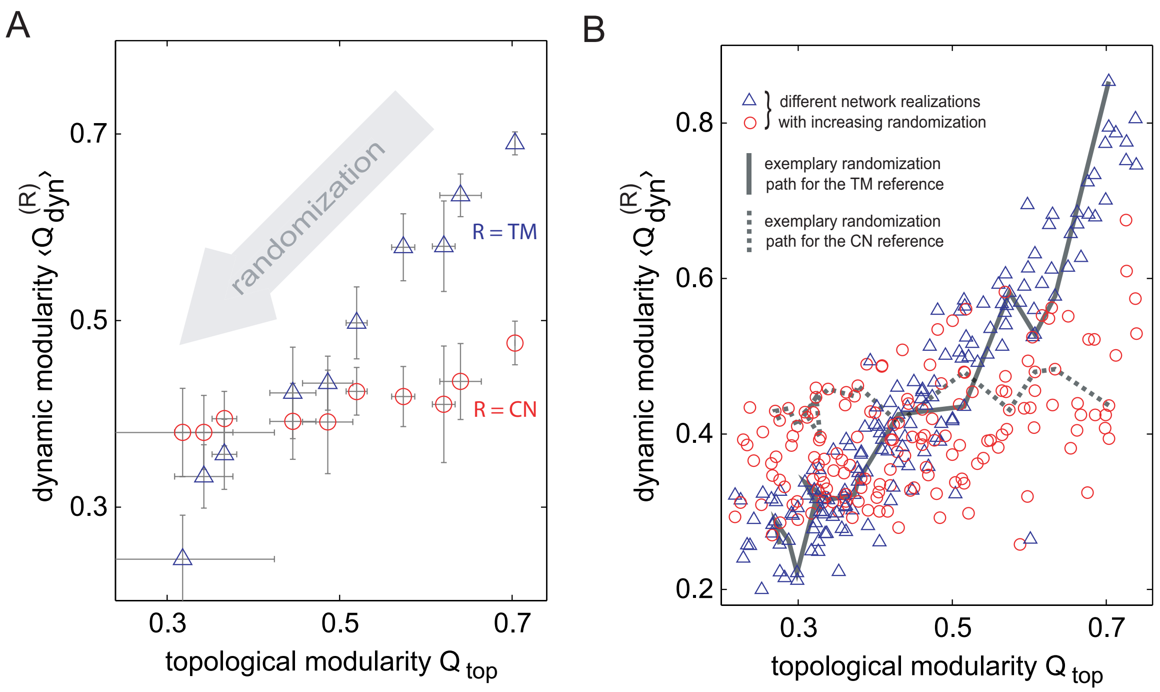

Figure S 1: Computation of the average dynamic modularity $\langle Q_{dyn} \rangle$ as a function of the topological modularity Q_{top} for different network realizations of the modular scale-free graph. Depicted are the TM results (blue Δ) which have been averaged over the range of $0.01 < f < 0.1$ and the CN results (red ○), averaged in the respective range of $10^{-6} < f < 10^{-5}$. The modular graphs ($n = 250$, $m \approx 515$ with $m_E = 3$) were randomized in several steps producing networks with similar graph statistics but a decreased modularity. (A) Average randomization path of 10 different randomizations of the same network. The strong correlation between the TM dependent values of $\langle Q_{dyn} \rangle$ and the topological modularity Q_{top} proves the assumption that this level of dynamic organization has to be regarded as a consequence of the particular exploitation of modular network structures via burst dynamics. The respective exploitation via spike dynamics remains small and comparatively constant. (B) A similar behavior is also true for different network realizations and their respective randomization paths. These networks display the same correlation between $\langle Q_{dyn} \rangle$ and Q_{top}. One randomization path from A has been highlighted.

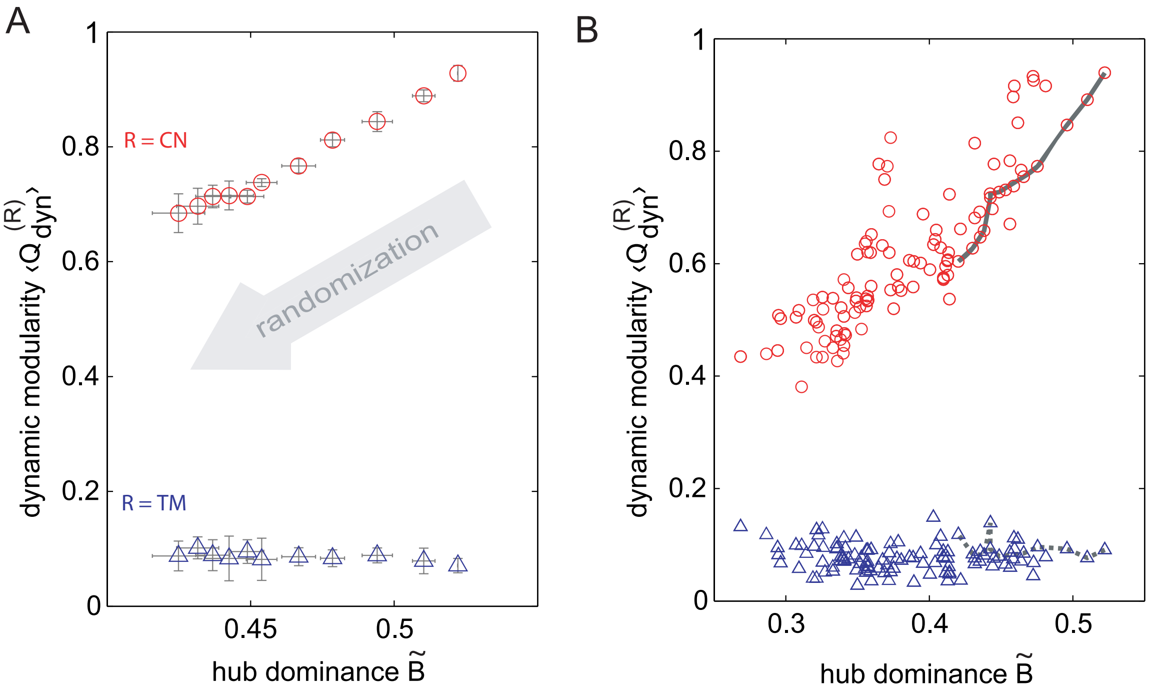

Figure S 2: Computation of the average dynamic modularity $\langle Q_{dyn} \rangle$ as a function of the hub dominance $\widetilde{B}$ for different network realizations of the BA graph. Corresponding to Fig. S 1A and B, different BA graphs and their randomized versions have been examined. (A) The randomization procedure causes a decrease of the hub dominance and, accordingly, a reduction of the CN-dependent values of $\langle Q_{dyn} \rangle$. These results confirm the assumption that the whole graph structure and the central node in particular are responsible for the emergence of ring-shaped excitation waves, whose regularity is more and more disturbed with increasing randomization steps. (B) The randomization versions of the different networks are separated across the decreasing curve, but show nevertheless the same correlation as in A.

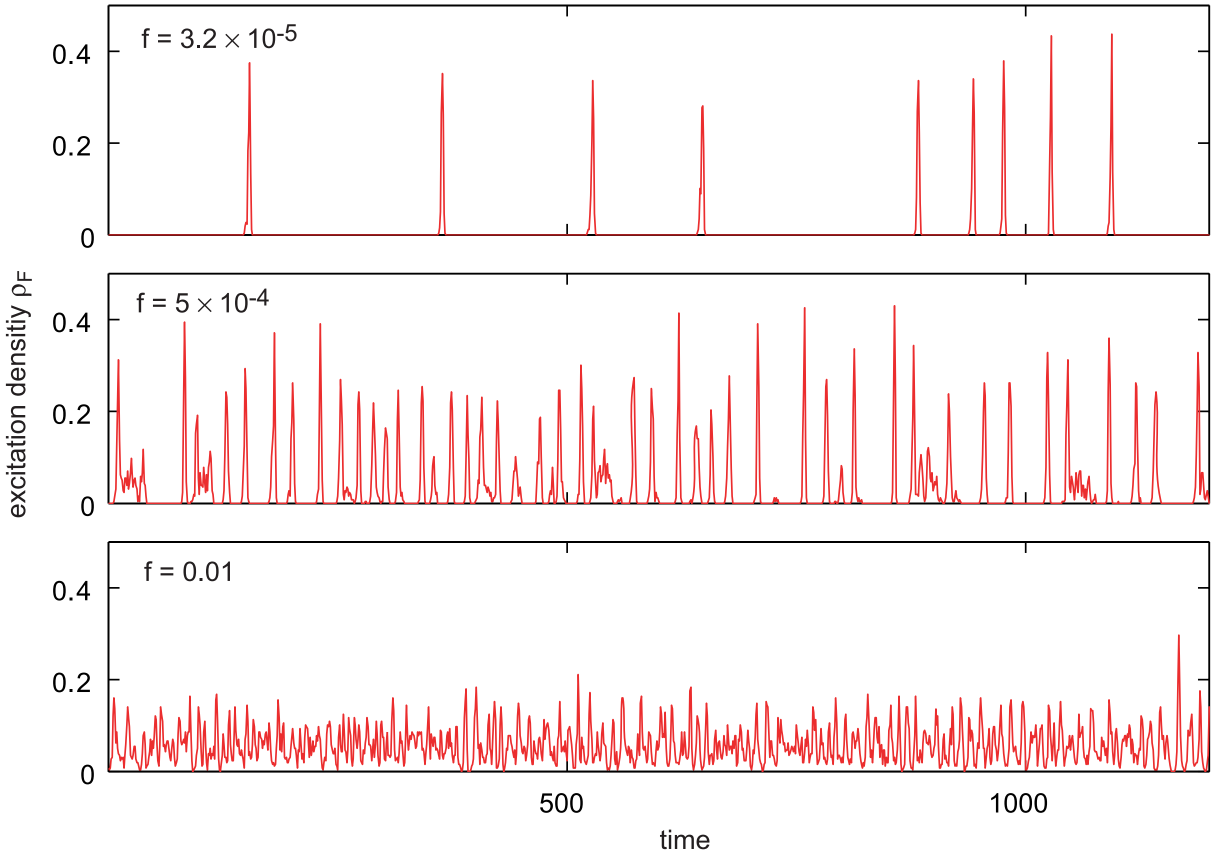

Figure S 3: Sections of time series of the excitation density ρ_F of the hierarchical scale-free graph (see Methods) for different rates of spontaneous excitations f. (From top to bottom) Increasing parameter f induces a change of the dynamic behavior from spike dynamics to burst dynamics with a transition region of f displaying a mixture of both dynamic regimes.

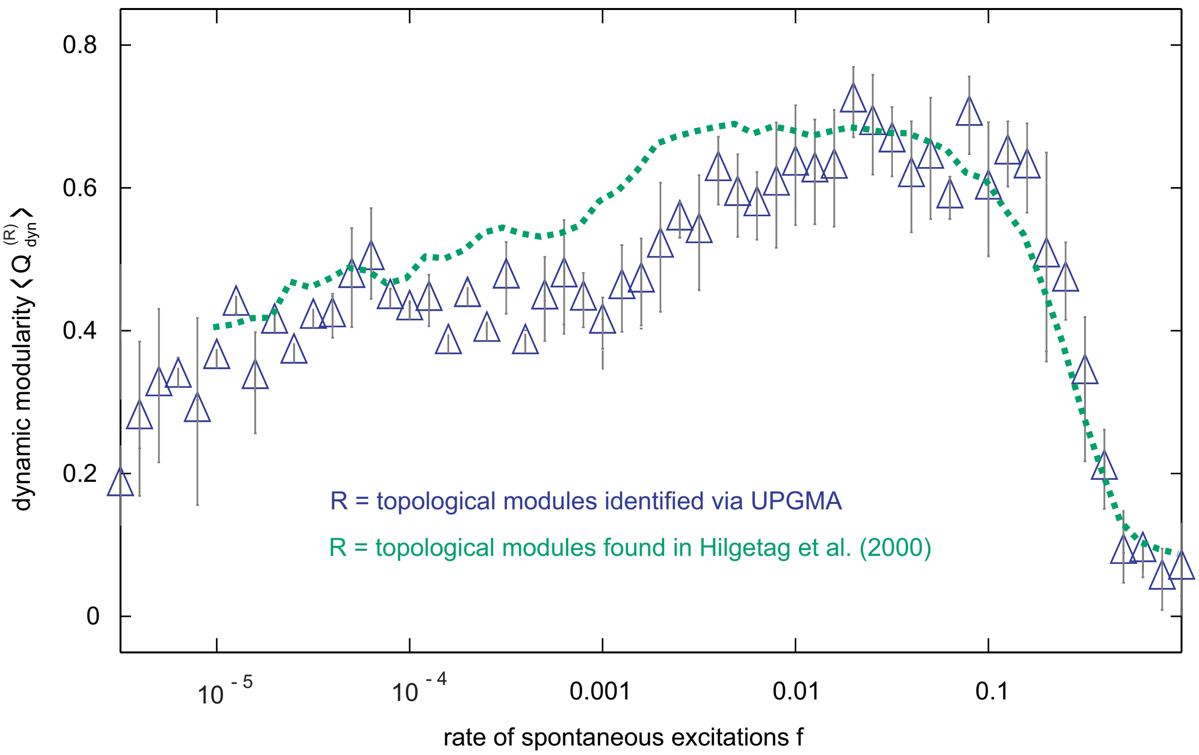

Figure S 4: Dynamic organization within the modular structure of the cortical network of the cat for two different definitions of the individual module composition. The curve indicated by the blue triangles corresponds to the TM-dependent results obtained from a UPGMA cluster analysis of the graph's distance information (using a threshold for 4 modules; see also Fig. 9A). Similar results (green curve; errors are of the size of the other results) were obtained from simulations using a different TM-reference consisting of 5 modules which have been identified in a work of Hilgetag et al. (2000). The additional module contains three nodes which could not be assigned to the remaining modules. Concerning the individual composition both references display a high consistency (75%).

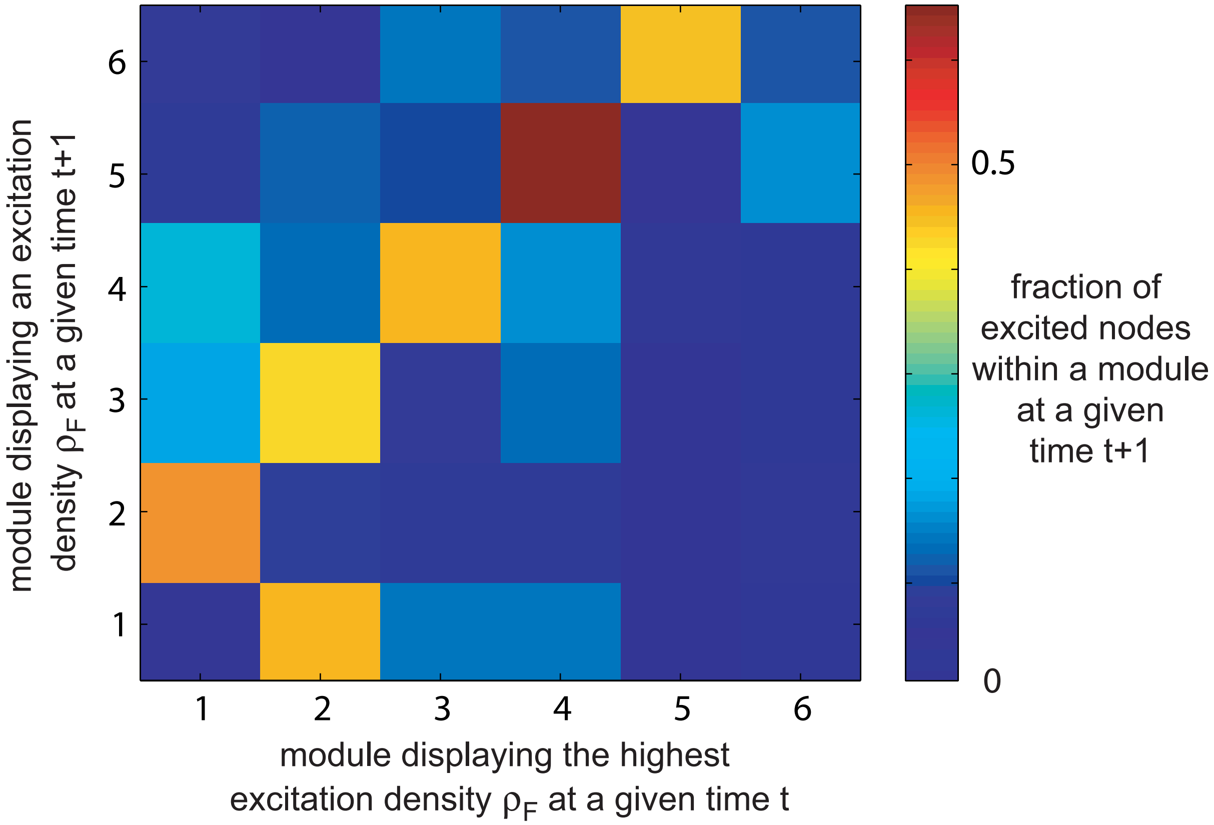

Figure S 5: Average fraction of excited nodes within each module resulting from previous excitations in a simulation of the scale-free (BA) graph in the spike-regime ($p = 0.1$ and $f = 10^{-4}$): In the presence of excitations within the BA graph at a given time t the respective module (which is the concentric arrangement of nodes resulting from the CN-reference) with the strongest excitation density ρ_F, i.e. the biggest fraction of excited nodes compared to its module size, has been identified. As a function of this module (the numbers on the abscissa denote the distance of the modules to the central node) the distribution of excitations over all modules has been computed for the following time step $t + 1$ and depicted on the ordinate as the module-specific fraction of excited nodes. Based on the central node and the resultant concentric modules there is an apparent propagation of the excitations in the spike-regime from the center of the graph to its periphery including an average module-specific excitation of 45 to 65 percent of the respective nodes.
